# Ergoapiol (Smith) in Diseases of the Female

**Published:** 1902-08

**Authors:** Charles H. Shepard

**Affiliations:** Physician to Lincoln Hospital, Dunham. N. C.


For Texas Medical Journal.

### Ergoapiol (Smith) in Diseases of the Female.

BY CHARLES H. SHEPARD, M. D.,
Physician to Lincoln Hospital, Dunham, N. C.

A deep and general interest is attached to all knowledge pertaining to the treatment of common diseases of the uterus, to which women are subject, and a vast literature is the outcome of this profound and focussed interest. We live today in an age of transition —a period of change. A great many of the former theories in medicine are fast passing away. New medicines are made, achieve a short-lived success, and then pass on to obscurity. This is true most especially in medicines for gynecological diseases. Of the newer remedies it is hard, indeed, to get one that may be depended upon for long. They soon lose their reputation and potency and are relegated to the past.

We know that all diseases of the womb have not the same etiology nor the same pathology; therefore, they should not all have the same treatment. Far too often the general practitioner groups all these diseases together as one and gives the routine treatment. It is not enough to give anodyne medicines for dysmenorrhea any more than it is sufficient to treat alike all forms of dysmenorrhea.

The operation of curettement has a most important place in these conditions, but like other remedial agencies it has its limitation. When we curette the uterus we rid it of a pathologically obnoxious lining membrane, and afford a normal membrane the opportunity to be formed.

The healthy woman with normal genitalia menstruates regularly and painlessly once a month from puberty to the "turn" of life, except that this regularity is interrupted by pregnancy and afterwards by lactation. Any departure from this rule constitutes an abnormality. Amenorrhea is less frequently met with than dysmenorrhea and irregular menstruation. The present age of transition has brought forth what is popularly known as the "new woman," and she has brought with her new ideas and practices which in very many cases retard growth and the natural process necessary for perfect health. For leaving the old landmarks, she has to suffer.

The most generally useful medicine in the condition of amenorrhea, dysmenorrhea, irregular, scanty and fetid menstruation, in my judgment, is a preparation of the Martin H. Smith Company, of New York, known as Ergoapiol (Smith). In the female ward of the Lincoln Hospital, Durham, N. C., I have used this medicine very extensively, and it has not only never failed to benefit and cure, but I know no remedy with which I could replace it were I deprived of it. Its efficacy may be tested by any physician who properly tries it. I mention a few cases, with short description of each, in which it has given the most signal benefit in my hands.

Ergoapiol (Smith) is put up as a small capsule, and is made up of a special form of apiol which is of the very highest quality. Combined with this are some other most valuable hemagogues, and they all go to make a fine preparation. It seems to be a scientific pharmaceutical preparation, non-toxic, tonic, as well as emmenagogue. What I have to say of this preparation is based entirely upon clinical experience, and I feel safe in saying that it will bear a clinical test whenever properly administered.

REPORT OF CASES.

CASE No. 1. Mrs. F. was admitted to hospital September 15, 1901; married; no children, though she had been married four years. Had not menstruated for seven years. Womb had been curetted several times; suffered from leucorrhea; pains in right and left iliac regions continuous. Examination showed a very small os, but generative organs were otherwise found to be normal. Another curettement failed to bring on the menses. I then prescribed Ergoapiol (Smith) to be taken one globule three times a day, and afterwards increase to one globule four times a day. After seven days of this treatment she complained of a general feeling of stiffness in her limbs, gaping and a feeling of malaise. The following morning she found to her delightful surprise that she was menstruating for the first time in seven years. At that time the flow was somewhat scanty, but the treatment was continued through three periods. Each succeeding period was more nearly normal than the one that preceded it. Now her functions are regular, and I know no reason why she may not become pregnant.

CASE No. 2. Mrs. S. complained of a continuous, dull, dragging pain, situated in the region of the iliac fossa of the right side. Menstruation irregular, scanty, fetid. Married six years; had never been pregnant. Excessive leucorrhea, though otherwise she was perfectly normal. Her weight was 140 pounds. Her condition, and the suffering, both physical and mental, which it occasioned her, was rapidly undermining her health. She was becoming emaciated, appetite of no consequence, general weakness. She considered her condition "hopeless." Cardiac weakness, of which she was a victim, contraindicated curettement—which usually cures "whites" and allows the formation of a healthy lining membrane. Ergoapiol (Smith) was prescribed for her, one capsule three times a day. In conjunction with this I gave tonic medicines. After six weeks' use of this remedy, the woman said she was "feeling so good" that she did not need any further treatment. She had increased in weight, and her appetite had become all she could wish. The menstrual flow was increased, and now, five periods having elapsed from the time treatment was instituted, her monthly flow has failed to appear. She does not expect its return for some time —supposing herself pregnant.

CASE No. 3. Miss S. suffered severe pain each month beginning a day before the flow came on. The flow was a thick, clotted mass, consisting of menbrane and the menstrual blood matted together. She had suffered from puberty, and the suffering became more intense as the years passed on. She was 19 years of age, stout, of healthy parentage. Admitted to Lincoln Hospital January 15, 1902. She declined an operation. I afterwards prescribed Ergoapiol (Smith) and have continued it for one month. Her next menstruation was free and easy; painless and regular. I doubt not that keeping up this treatment up to another period she will be entirely rid of the hitherto troublesome condition.

Case No. 4. Miss W. Tubercular history. Menstruation very irregular, sometimes three, sometimes five, weeks between periods; very painful; scanty. I prescribed Ergoapiol (Smith) one capsule four times a day beginning one week before the menstrual period and continued a week after the period. As a result of this treatment the patient feels a great deal better in her general health; her monthly flow has been rendered painless and increased in quantity. Ergoapiol has a tonic action upon the muscular fibers of the womb. Its effect is not transitory, but lasting. This superior preparation is decidedly tonic.

Case No. 5. Mrs. D. A victim of endometritis. Pain continues between periods and is aggravated at periods. Leucorrhea was very pronounced; pains in the back; "hot flushes"; vertigo; headache. Patient would not allow an operation; highly sensitive. Several preparations were tried, but none gave relief until Ergoapiol (Smith) was used. It has entirely relieved the patient, and she is now loudly singing its praises. In this case treatment was kept up for ten weeks.

Ergoapiol has never failed in my hands. It is not possible that it can cure obstructive dysmenorrhea, but with that exception it is indicated in all the other diseases of the womb where a tonic and sedative action is the requirement.

Case No. 6. Mrs. D., widow; aged 33; had three children; youngest 10 years of age. She had suffered all her menstrual life severe pains in the pelvis at each period; had to keep in bed a week or more each month; paroxysms of pain were followed by a flow of the "whites"; no anemia; womb found to be flabby and relaxed; pains extended down thighs posteriorly. Had been treated for many years by various physicians of note, but had received only temporary benefit. Ergoapiol (Smith) was given her, one capsule three times a day, and increased at the time of the flow to four a day. After three months of this treatment her menstrual function became regular, and being entirely well now, she feels that life, after all, is worth living.

I could prolong this list indefinitely with records of cases that have been entirely relieved of these conditions, and I shall be pleased to furnish any information desired as to Ergoapiol (Smith) and its use.

